# A comparison of referral and treatment patterns at child development institutes during the corona closures and pre-corona periods

**DOI:** 10.3389/fpubh.2025.1447000

**Published:** 2025-07-18

**Authors:** Hallel Katsir, Michael Davidovitch, Michal Elboim-Gabyzon

**Affiliations:** ^1^Department of Physical Therapy, Faculty of Welfare and Health Science, Haifa University, Haifa, Israel; ^2^Child Development, Medical Division, Maccabi Healthcare Services, Zichron-Yaakov, Israel

**Keywords:** referral, treatment, child development, corona, closure

## Abstract

**Background:**

During the COVID-19 pandemic, there was a marked shift in the factors affecting health behaviors, suggesting a potential change in the rate of referrals to child development institutes during this period. To date, the rate of referrals to child development centers during the pandemic has not been thoroughly examined.

**Objective:**

To examined the difference in the annual and monthly referral and treatment patterns at child development institutes in the year 2020 (corona lockdown year) compared to the referrals in the year 2018–2019 (pre-corona period).

**Methods:**

This retrospective cohort study analyzed data from the medical records of applicants to nine Child Development Institutes operated by the second-largest health fund in Israel (Maccabi Health Services). Descriptive statistics (medians and IQR for continuous variables, frequencies and percentages for categorical variables) were calculated for the pre-COVID period (2018–2019) vs. the COVID-closure year (2020). Inferential analyses used chi-square or Fisher’s exact tests for categorical outcomes and Mann–Whitney tests for continuous outcomes.

**Results:**

During the COVID-19 closure period in 2020, there was a slight decrease (2.77%) in the annual number of referrals compared to the average of the preceding years 2018–2019 (13,143 inquiries and 13,518 inquiries, respectively). This decrease occurred primarily during the first lockdown period (March–May, 2020). The percentage distribution of annual diagnoses across various healthcare professionals was consistent across all periods examined. Notably, a substantial increase in the percentage of remote treatments was observed during the closure period compared to the pre-COVID era. Differences emerged in the demographic and social characteristics of the applicants during the closure period, such as their socioeconomic status and age.

**Conclusion:**

COVID-19 closures altered referral patterns to child development centers in both volume and applicant characteristics. Caregiver concerns about infection risk reduced in-person visits; restrictions changed clinic accessibility and drove rapid telehealth adoption; and staffing reassignments modified referral protocols. As a result, initial referrals declined, remote treatments increased, and applicant profiles skewed younger with shifts in socioeconomic and insurance status. To maintain continuity during future disruptions, hybrid delivery models, robust telehealth infrastructure, and streamlined appointment processes are essential. These insights can also inform prevention programs, policy planning, and resource allocation during public health crises.

## 1 Introduction

Individuals seeking health services display complex health behaviors influenced by factors at three levels: individual, community, and healthcare organization, grounded in Andersen’s behaviorist model (1968) (1), and McLeroy and colleagues’ socioecological model (1988) (2).

An extensive knowledge base has been developed regarding COVID-19, which has been thoroughly investigated globally across various medical disciplines. The focus has been primarily on infection dynamics, symptoms, and their impact on diverse healthcare professionals (3–7). Notably, the influence of the coronavirus on referrals to child development centers remains unexplored, both on a global scale and within Israel. At the individual level, COVID-19 has impaired the motor and social experiences of children, potentially causing global developmental delays (8–28). These studies underscore a substantial lifestyle shift with challenges in social and mental dimensions, altered sleep patterns, fluctuations in physical activity, and increased sedentary behavior. These changes are expected to boost demand for child development health services.

Referrals to child development centers diverge from other medical services because of the child’s age, hindering the independent application of medical services. The primary referrer to child development centers is invariably the parent. Information regarding children’s parental functioning during the Corona period assumes critical importance given the pivotal role parents play in their children’s cognitive and emotional–social development, shaping an enriched learning environment (13). On the one hand, compared to the pre-corona period, there has been a substantial increase in parent–child time during COVID-19, resulting from extended periods spent at home due to school and kindergarten closures, alongside some parents transitioning to remote work (13, 29, 30). On the other hand, a notable manifestation of negative factors occurred: the corona epidemic adversely affected mental state of parents, leading to a notable increase in states of sadness, depression, and anxiety (13–15, 31, 32).

This has resulted from an escalation of stress factors for parents, including low family income, unemployment, and concerns about their own and their children’s health (33). In some cases, parents’ mental state has been cited as a contributor to the adoption of negative educational strategies and unfavorable behavior toward their children. Another unique characteristic of this period that may decrease referrals to children’s centers is the fear among parents of infants seeking preventive medicine because of concerns about exposure to and infection from the coronavirus in the community (31). This concern was particularly pronounced among parents who used public transportation (31). Additionally, fear of viral exposure has led to social isolation of children, with parents avoiding gatherings with other children (32).

At the organizational level, substantial changes occurred in the work patterns of the public health system in Israel during the corona period, particularly among the employees of child development institutes (34). These employees divided their working time between onsite work at the institute and remote work facilitated by video/zoom calls and telephones. For instance, at the child development institutes of Israel’s largest public health fund, caregivers were directed to prioritize remotely guided treatment. Patients visiting the fund’s branches were instructed to come to the institutes with a single companion, excluding siblings, and wear masks (34).

Due to restrictions on gatherings in Israel, informal organizations supporting maternal and infant health were forced to close, reducing face-to-face services and potentially lowering parental awareness. This decline may have contributed to a decrease in referrals to child development centers. Conversely, an increase in referral rates is expected among populations distant from service centers or those reluctant to visit because of COVID-19 constraints. This shift is attributed to health funds providing community services, including child development centers, through telemedicine—encompassing diagnosis, treatment, and training via phone, video, or online platforms, such as Zoom or email (35, 36). Our hypothesis is supported by a study conducted in the United States that demonstrated a significant surge in referrals to health services for eating disorders among teenagers during the Corona period. This increase was attributed to the transition to telemedicine, a service that facilitates access for individuals residing away from treatment centers (35). However, contrasting evidence from the United States also indicates reduced utilization of telemedicine services among low-income individuals and members of ethnic minority groups during the same period, primarily due to economic considerations (6, 7).

As mentioned, there is a notable gap in the examination of the consumption patterns of services provided by child development institutes during the Corona period, a topic that has yet to be studied both globally and in Israel.

## 2 Objectives

- To investigate the annual and monthly service referrals from various pre-medical care professionals in child development institutes in Israel during the COVID-19 closures in 2020 and compare them with the referrals in the preceding 2 years (2018–2019).
- To explore the number of diagnoses, the referral-to-diagnosis times, and the number and types of treatments at child development centers during the COVID-19 closures in 2020 in comparison to those in the years 2018–2019.
- To explore the demographic, social, and familial characteristics of individuals seeking services in child development centers during the COVID-19 closure period and before the pandemic.

## 3 Materials and methods

This retrospective cohort study analyzed data from the medical records of applicants to nine Child Development Institutes operated by Maccabi Health Fund) the second-largest health fund in Israel(, covering approximately 22% of all such institutes nationwide. The study period spanned January 2018 through December 2020, allowing comparison of two full pre-COVID years (2018–2019) with the COVID-closure year (2020). Applicants up to 6 years of age were included.

### 3.1 Outcome measures

We categorized outcomes into general and personal measures:

#### 3.1.1 General outcome measures

- Referral counts: The date of “referral” was defined as the date on which a referral request was received by the Child Development Institute. Annual and monthly numbers of such referrals to each Child Development Institute, stratified by field of care (physiotherapy, occupational therapy, communication clinics, developmental psychology, developmental medicine, and social work) was recorded.
- Diagnosis counts: Diagnosis” was defined as the first confirmed clinical evaluation outcome, in which a qualified specialist identified the child’s developmental or behavioral condition. The date of “diagnosis” was defined as the date on which a qualified specialist rendered a confirmed clinical evaluation outcome, identifying the child’s developmental or behavioral condition. We recorded the total number of such diagnoses in each field during 2018, 2019, and 2020.
- Number of treatment modalities: Treatment” was operationally defined as any therapist-delivered clinical intervention provided by a qualified professional, including speech therapy, occupational therapy, physical therapy, developmental psychology sessions, and developmental medicine consultations. These treatments are categorized into face-to-face and remote modalities, encompassing phone-based or remote video sessions, but it was not feasible to distinguish between different types of remote treatments.
- Referral-to-diagnosis time (waiting time): Calculated as the number of days between the referral date and the diagnosis date for each child. This “referral-to-diagnosis” interval was recorded for all cases with complete date information.

These general measures were tracked for both the COVID-closure period (March–December 2020) and the combined pre-COVID period (January 2018–December 2019).

#### 3.1.2 Personal outcome measures

- Demographic and social factors: Patient’s date of birth (used to calculate age), sex, place of residence, number of children in the household, and the birth order of the applicant.
- Administrative details: Date of first assessment at the institute; Date of formal diagnosis by applicable professionals (e.g., developmental pediatrician, psychologist); Number of treatments received by each patient; Referring party (health-fund physician vs. hospital); Referring physician’s specialty.
- Medical diagnoses: Each diagnosis assigned by the developmental pediatrician at the institute was coded into one of approximately 260 codes (some administrative, e.g., “monitoring,” “form completion,” and some clinical). For inferential testing, we aggregated these into 31 clinically meaningful groups, focusing on those likely influenced by service access: communication disorders, behavioral difficulties, attention/concentration issues, developmental delay, and similar categories.
- Socioeconomic status (SES): Determined by the Central Bureau of Statistics settlement ranking, where a ranking of 1–4 denotes low SES, 5–7 denotes medium SES, and 8–10 denotes high SES.
- Insurance level: Maccabi insurance tiers 1–4 = basic, 5–8 = medium, 9–12 = high. To capture a representative snapshot of the pre-COVID era, the period of COVID-related closures and the 2 years preceding them were compared. This approach was chosen to avoid presenting the atypical situation that a single year might depict. This method is consistent with the results of previous studies (6, 8). The year marked by the closures was selected to characterize the COVID period.

### 3.2 Descriptive statistics

Descriptive statistics for all outcome measures across the three calendar years were first calculated:

- For continuous variables (e.g., monthly referral counts, waiting time; days from referral date to diagnosis date), and (total number of treatments per patient), we report medians and interquartile ranges (IQR) because these distributions were non-normal. We also adjusted referral counts by the total number of Maccabi-insured children aged ≤ 6 years in each month and year, to account for annual fluctuations in the eligible population denominator.
- For categorical variables (e.g., referral source, age group [0–1 years, 2–3 years, 4–6 years], sex, SES category, insurance tier, and treatment modality), we report frequencies and percentages.

To avoid basing conclusions on a single pre-COVID year (which can be atypical), we combined 2018 and 2019 as the “pre-COVID period” and compared these 2 years to 2020 (the “COVID-closure period”). This approach follows precedent in similar studies (6, 8) and provides a representative snapshot of baseline referral and treatment patterns.

### 3.3 Inferential analyses

After visualizing descriptive trends, we performed inferential tests to determine whether observed differences between the pre-COVID period (2018–2019) and the COVID-closure year (2020) were statistically significant:

1. Categorical outcome variables, including referral source, age group, SES category, insurance level, treatment modality, were compared across the three individual years (2018 vs. 2019 vs. 2020) using chi-square (χ^2^) tests. Where cell counts were low (< 5), we applied Fisher’s exact test.
2. Continuous variables with non-normal distributions, specifically: Monthly referral counts (in each treatment field); Referral-to-diagnosis time- waiting time (days between referral date and diagnosis date) and total number of treatments received per patient were compared across the 3 years using the Mann–Whitney test. Statistical significance was defined as *p* ≤ 0.05. All statistical analyses were conducted using SAS version 9.4 (SAS Institute, Cary, NC, United States).

## 4 Results

### 4.1 The number of annual and monthly referrals to child development institutes

**Figure 1:**
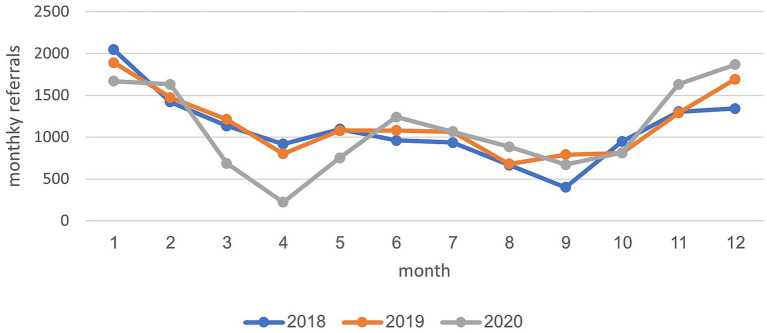
Monthly diagnosis rates from all referrals to child development institutes across the years 2018–2020.

The annual and monthly referral data for child development institutes are presented in Table 1 and Figure 1. The analysis revealed that during the period of Corona closures (1.2020–12.2020), the annual number of referrals was 13,143, compared to an average of 13,518 referrals in the 2 years prior to the Corona pandemic (2018 and 2019). This reflects a decrease in referrals, equating to a 2.77% reduction. During the initial closure on March 14, 2020, lasting 2 months (March–May), there was a marked decline in monthly referrals by several hundred. Another substantial decrease in monthly referrals occurred in September 2020 by groups of 10 referrals, compared to those in the 2 years preceding this period.

**Table 1:** Monthly and yearly referral counts to child development institutes (2018–2020).

Year\month	2018	2019	2020
January	2047	1890	1,669
February	1,420	1,473	1,631
March	1,135	1,212	688
April	918	801	222
May	1,097	1,078	754
June	962	1,080	1,240
July	935	1,065	1,066
August	667	680	885
September	400	792	672
October	949	811	815
November	1,303	1,288	1,633
December	1,341	1,692	1868
Sum	13,174	13,862	13,143

### 4.2 Diagnosis rate during the review period (2018–2020)

**Figure 2:**
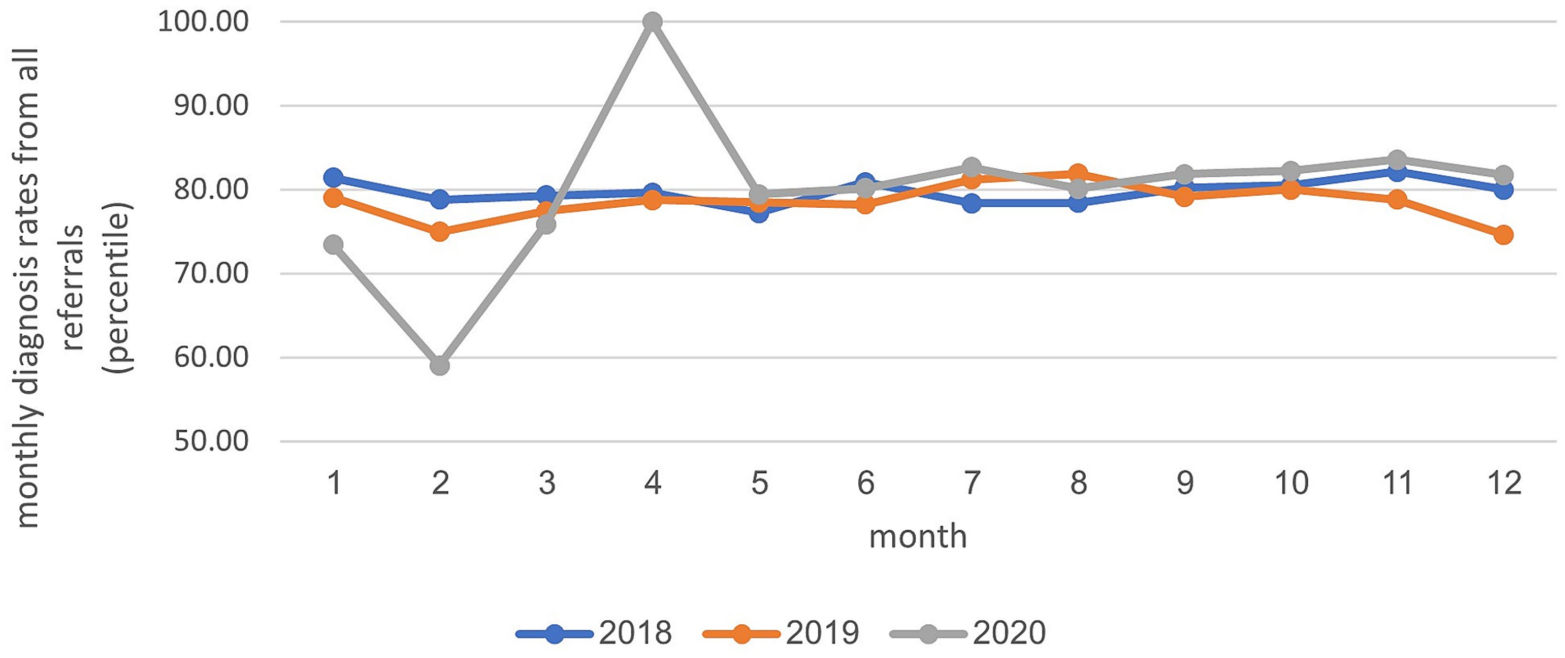
Distribution of annual treatments across diagnoses in child development institutes: analysis by examined age groups.

**Figure 3:**
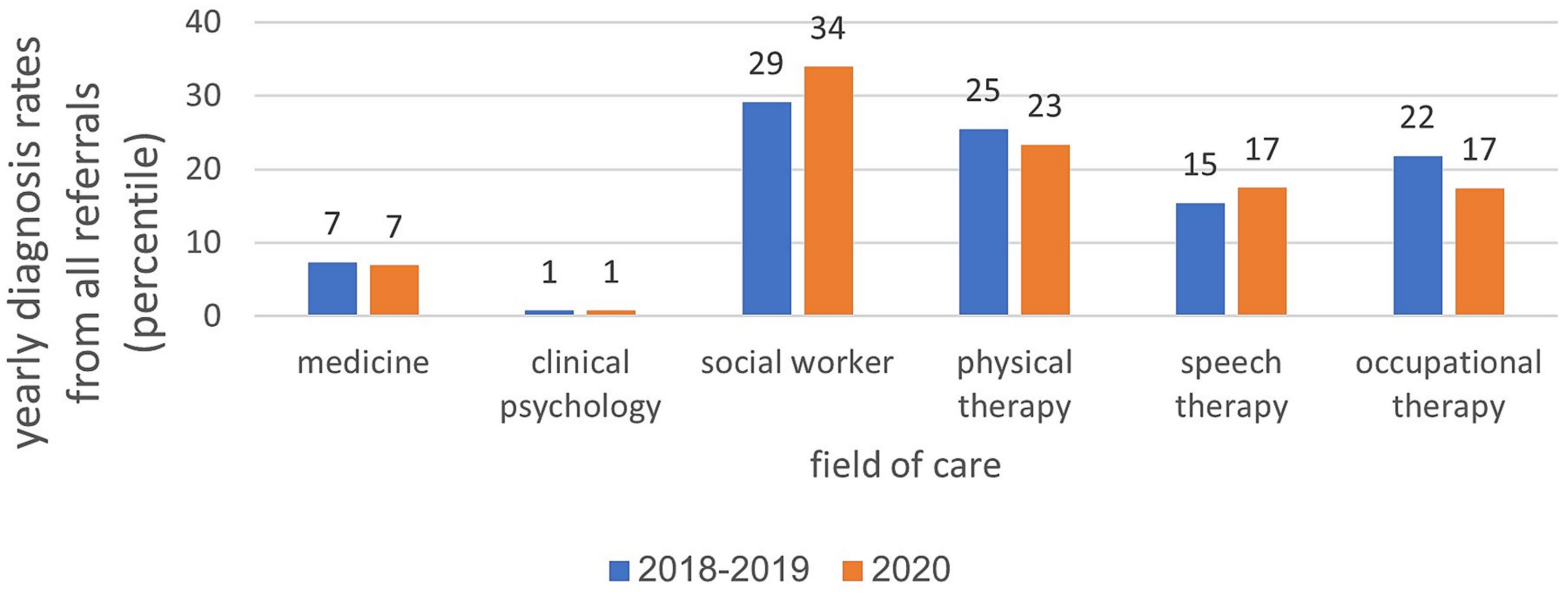
Distribution of annual treatments across diagnoses in child development institutes: analysis by treatment field.

In the years 2018–2019, an average of 10,680 diagnoses were recorded. In 2020, the average value decreased slightly, to 10,452. Figure 2 illustrates that the monthly diagnosis rate relative to all referrals to child development institutions across various treatment areas remained relatively stable, fluctuating between 69 and 83% during these years. Two distinct points in time are noteworthy: a sharp decrease (10–14%) in February 2020, just before the onset of the initial closure, and a notable increase (30%) in April 2020, coinciding with the closure period. Analysis of the annual diagnosis rates from all referrals to child development institutes categorized by areas of treatment revealed a consistent distribution across various treatment disciplines between 2018 and 2020. Most diagnoses were attributed to the healthcare professional staff at the institute, including occupational therapists, communication clinics, physical therapists, and social workers. During the COVID-19 closure period in 2020, there was a slight increase in the percentage of diagnoses in the fields of social work (5%) and communication clinics (2%), compared to diagnoses in the years 2018–2019. Conversely, there was a minor decrease in the percentages of diagnoses in the physical therapy (2%) and occupational therapy (5%) fields (see Figure 3).

### 4.3 Analysis by areas of treatment and annual treatment rate in child development institutes from 2018 to 2020

**Figure 4:**
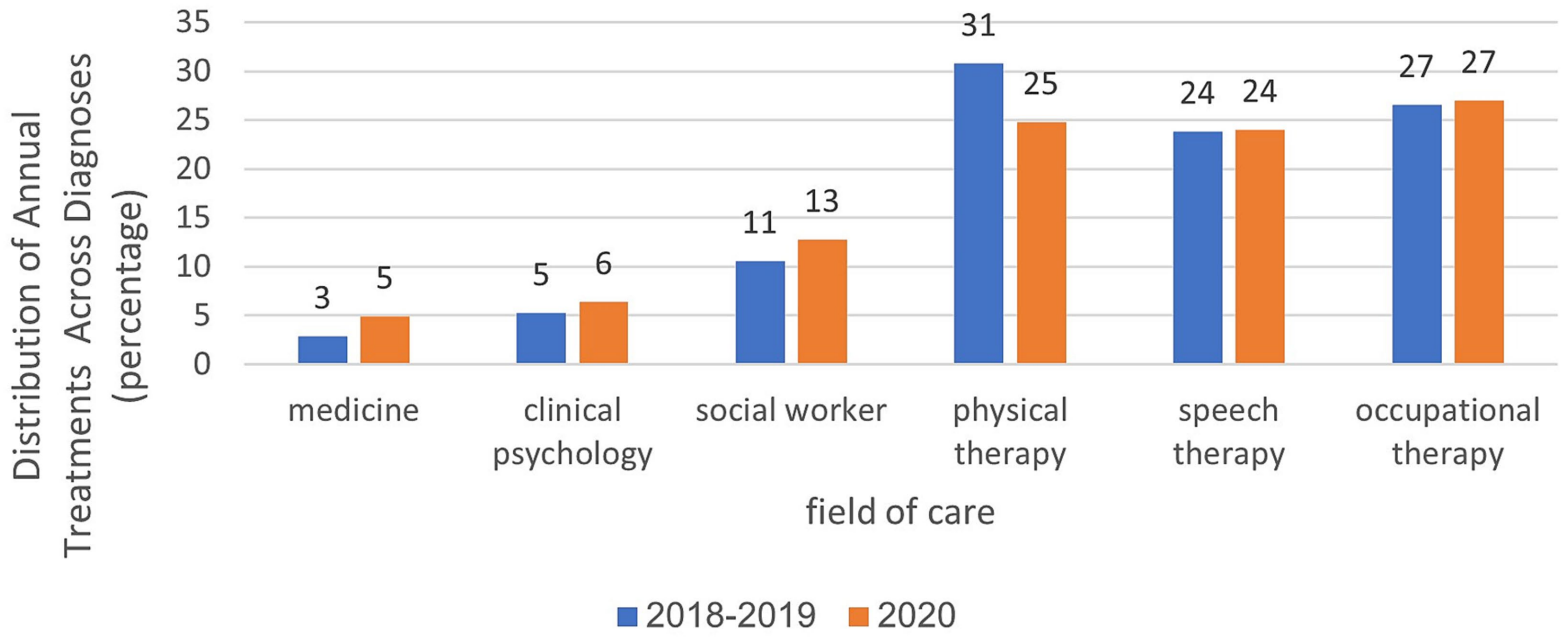
Analysis of treatment rates in age group 2–3 in relation to treatment areas and examined years.

**Figure 5:**
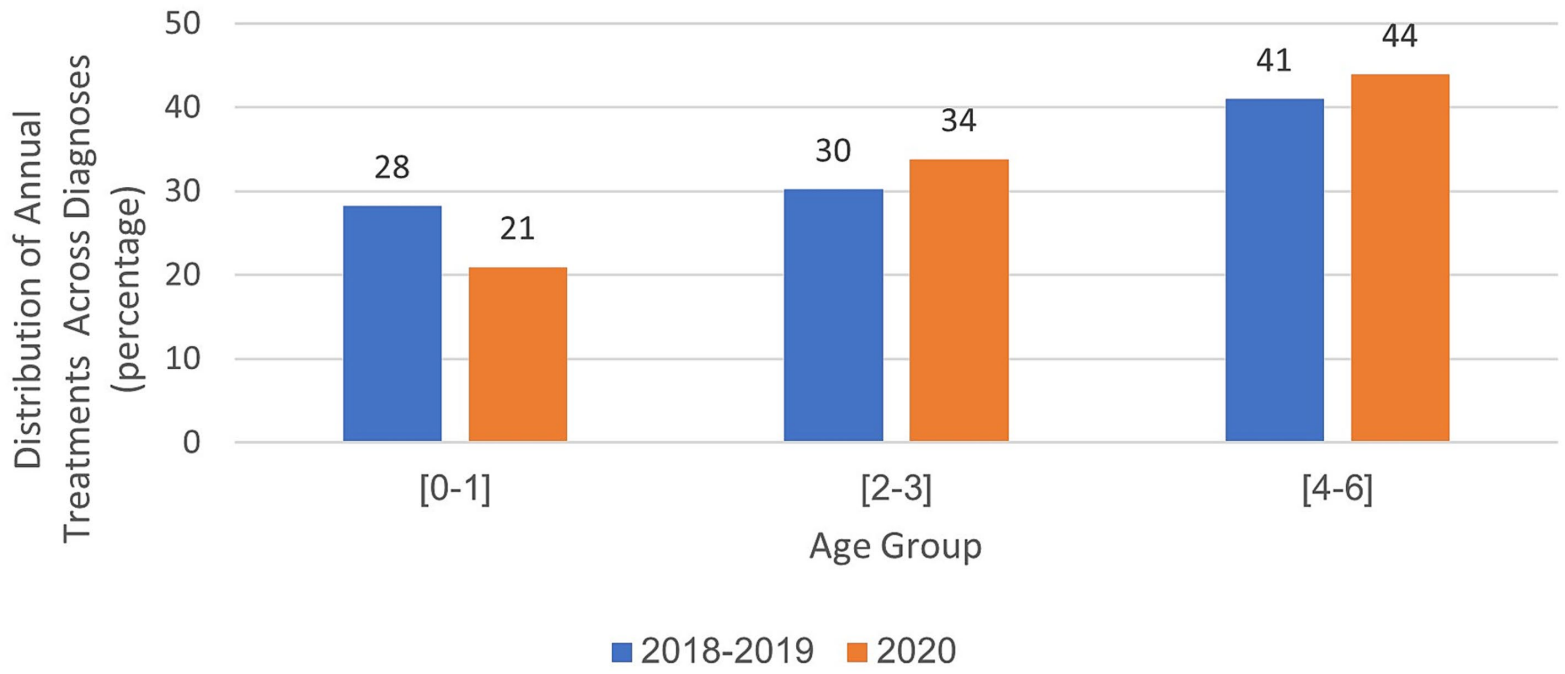
Monthly referrals to child development institutes (2018–2020).

In the years 2018–2019, an average of 56,033 treatments were administered. By 2020, this number increased to 89,648. The distribution of treatments across various healthcare professions remained consistent from 2018 to 2020, with a higher volume of treatments in paramedical fields, such as physiotherapy, communication clinics, and occupational therapy, than in social work and clinical psychology. In the field of physiotherapy, there was a 6% decrease during the year of closure (Figure 4). Analysis of the total annual rate of treatments across all diagnoses and distribution by the age of the children showed that in 2020, there was a 7% decrease in the 0–1 age group, a slight 4% increase in the 2–3 age group, and a 3% increase in the 4–6 age group compared with the period preceding the onset of the coronavirus. Further details are shown in Figure 5.

**Figure 6:**
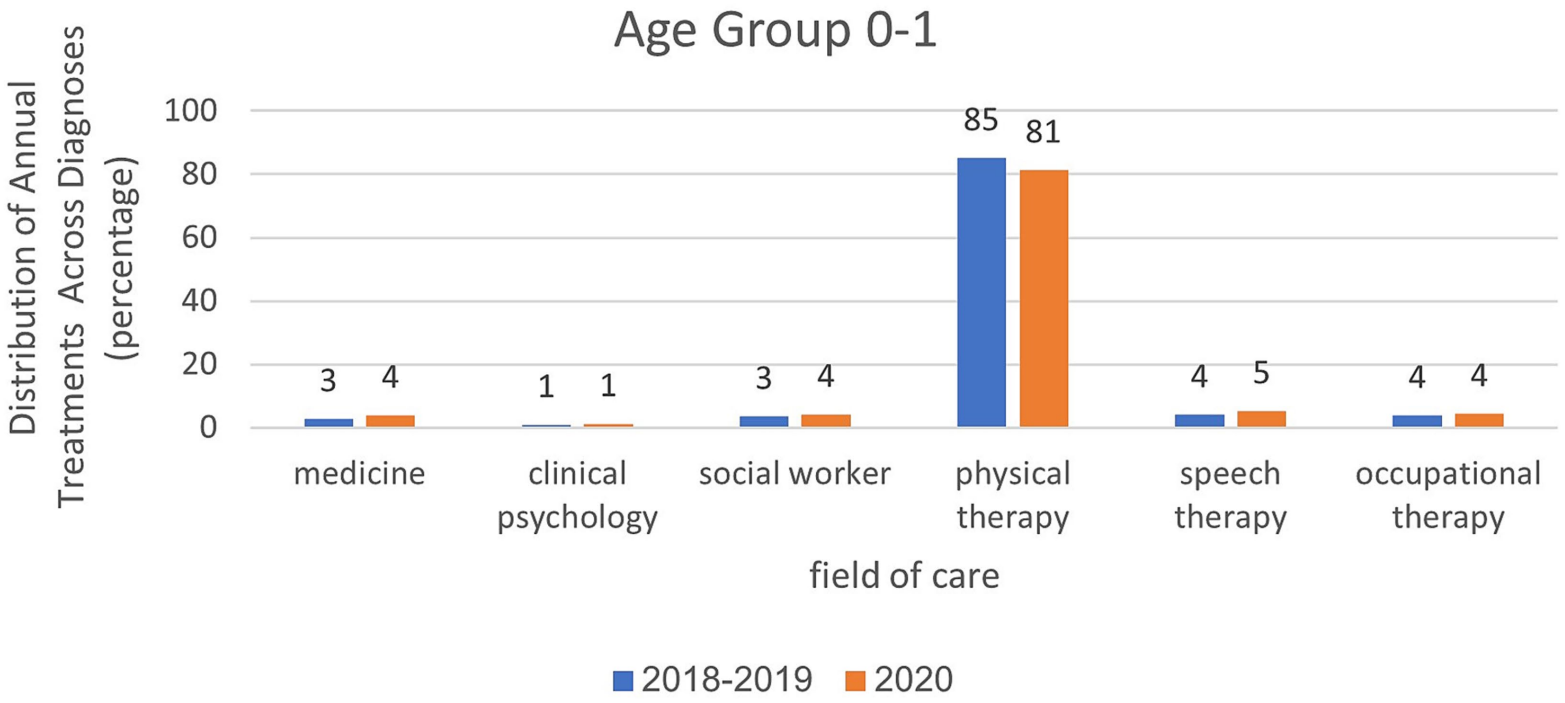
Analysis of treatment rates in age group 4–6 in relation to treatment areas and examined years.

**Figure 7:**
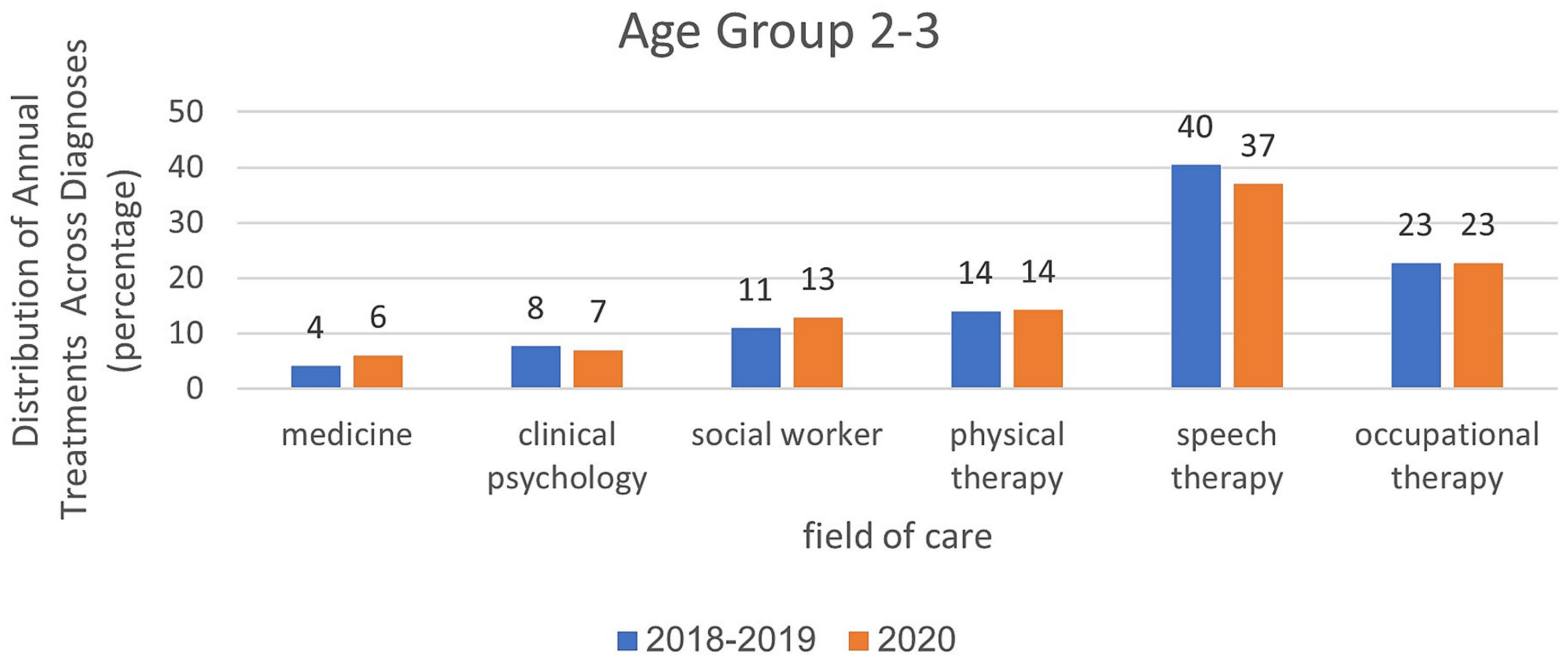
Analysis of treatment rates in age group 0–1 in relation to treatment areas and examined years.

**Figure 8:**
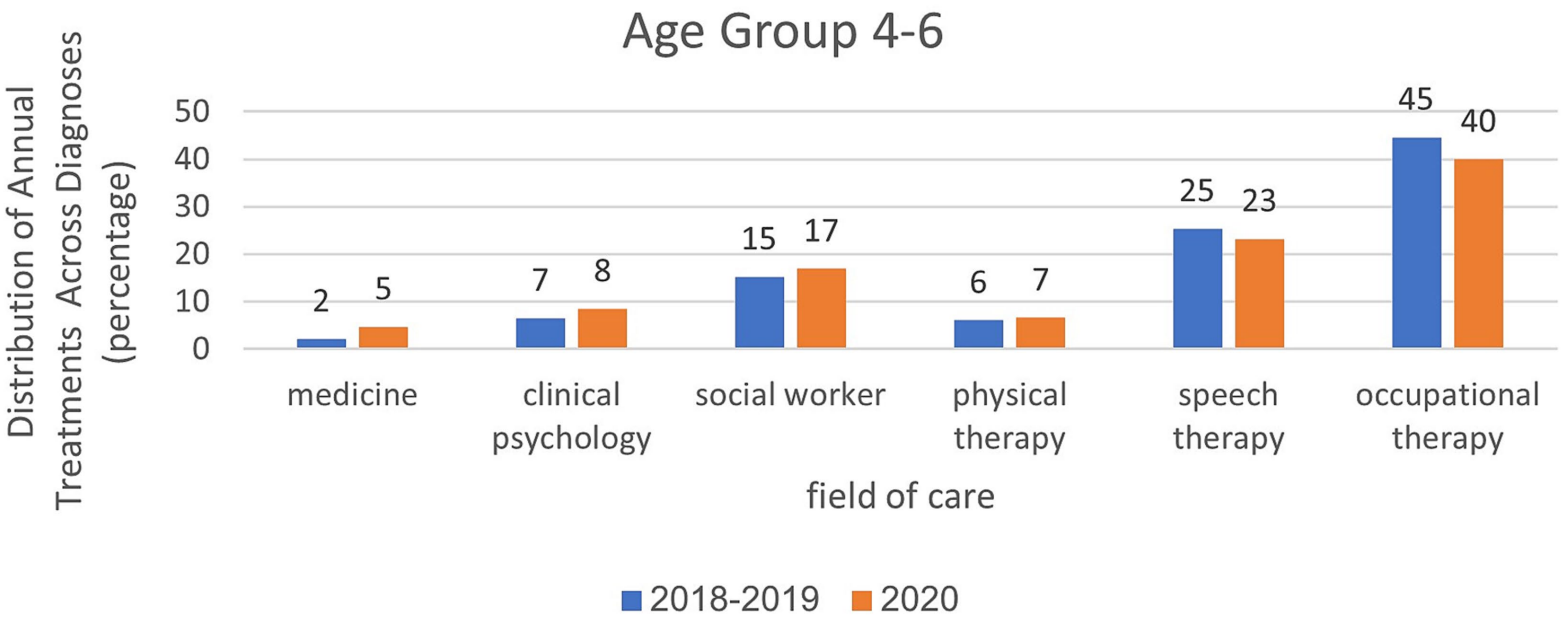
Proportion of diagnoses relative to all referrals to child development.

Analyses within each age group, categorized by treatment area and examined years, are presented in Figures 6–8. For the age group of 0–1 years, physical therapy accounts for the majority of treatments, with 81 to 85% of treatments as illustrated in Figure 6. For children aged 2–3 years, as depicted in Figure 7, a higher percentage of treatments occur in communication clinics (37–40%) and occupational therapy (23%). Conversely, for the age group of 4–6 years, Figure 8 illustrates that the highest proportion of treatments is found in occupational therapy (39–45%) and communication clinics (23–27%), consistently across the years 2018 to 2020.

In March–May, the data were similar to the annual data except that they were more prominent. For ages 0–1, the rate of physiotherapy treatments was 92% in the year of closures compared to 93% in the years before the corona virus. At the age of 2–3, the proportion of treatments was greater in the field of communication clinics and it was 29% in the year of closures, compared to 37% in the years before the corona virus. In the field of occupational therapy, the rate of treatment remains the same (20–21%). At the age of 4–6, the proportion of treatments was greater in the field of occupational therapy (34%) during closures than in the pre-Corona period (43%). In the field of communication clinics, the treatment rate ranged from 23% during the closure period to 25–26% in the pre-Corona period.

### 4.4 The number of remote treatments in child development institutes from 2018 to 2020

A notable surge in the annual absolute and relative rates of remote treatments across all age groups were observed to be 6.9% in contrast to 0.02% in the pre-COVID years. Specifically, the rate of remote treatments for ages 0–1 was 14.3%, surpassing the rates of 6.1% for ages 2–3 and 4% for ages 4–6, as detailed in Table 2.

**Table 2:** Annual quantity of remote treatments across years: absolute and relative analysis by age groups.

Year	All	Age 0–1	Age 2–3	Age 4–6	Age 7–9
2018 and 2019	20 (0.02)	7 (0.0)	6 (0.0)	7 (0.0)	0 (0.0)
2020	6,166 (6.9)	2,685 (14.3)	1859 (6.1)	1,576 (4.0)	46 (3.8)

Analysis of the specific rates of remote treatment from March to May revealed a noteworthy increase during the 2020 closure period. At all ages, there was a substantial increase in the rate of remote treatments, reaching 23.4%, in stark contrast to 0% in the years preceding closures. Notably, the age group of 0–1 year experienced an even more pronounced surge, reaching 40%. (Table 3).

**Table 3:** Monthly quantity of remote treatments (absolute and relative) in March–May, categorized by age groups.

Year	All	Age 0–1	Age 2–3	Age 4–6	Age 7–9
2018 and 2019	3 (0.0)	0 (0.0)	3 (0.0)	0 (0.0)	0 (0.0)
2020	2,805 (23.4)	1,183 (40.0)	954 (23.1)	650 (13.8)	18 (9.0)

### 4.5 Referral to diagnosis (waiting) time interval at the institute

The “referral-to-diagnosis” interval, defined as the number of days between the recorded referral date and the recorded diagnosis date, showed a statistically significant increase during the closure year (82.1 days), compared to the pre-coronavirus average interval (77.6 days, *p*-value<0.0001). For more detailed information, please refer to Table 4.

**Table 4:** Average waiting time (in days) from referral date to diagnosis date—using the Mann–Whitney Test.

Variable	Year	N	Mean	Std	Median	Minimum	Maximum	*p*-value
Average of days from acceptance of set to first diagnose	2018–2019	144	77.6	38.4	72.5	28.0	325.0	<0.0001
	2020	71	82.1	115.3	60.0	14.0	995.0	

### 4.6 Personal outcome measures

The demographic and social data of applicants to child development institutions during the years under review (2018–2020) are presented in Table 5.

**Table 5:** Demographic and social data of applicants to child development institutes from 2018 to 2022: an analysis using the “chi-squared test.”

Variable		2018–2019 *N* = 27,036 N (%)	2020 *N* = 13,143 N (%)	Significance
Sex (male/female)	Boys	17,114 (63.3)	8,258 (62.8)	0.093
Age group (years)	0–1	8,137 (30.1)	4,107 (32.2)	<0.0001
	2–3	6,245 (23.1)	3,535 (26.9)	
	4–6	12,649 (46.8)	5,501 (41.9)	
SES	Low 1–4	8,761 (32.5)	4,093 (31.2)	<0.0001
	Medium 5–7	11,492 (42.6)	5,843 (44.5)	
	High 8–10	6,735 (25.9)	3,190 (24.3)	
Insurance grading	Low 1–4	2,531 (9.4)	1,179 (9.0)	<0.0001
	Medium 5–8	13,107 (48.5)	5,499 (41.9)	
	High 9–12	11,398 (42.2)	6,463 (49.1)	

Demographic and Social Data: SES, socioeconomic status.

In the period spanning 2018–2022, the majority of applicants to child development institutes were males. No significant differences in the sex distribution were observed over time.

Notably, a significant decline (*p* value <0.0001) in the proportion of applicants aged 4–6 occurred during the closure period, dropping from 46.8 to 41.9%. Conversely, there was a statistically significant increase in the other two age groups (*p* value <0.0001): 23.1 to 26.9% for those aged 2–3 years and 30.1 to 32.2% (*p* value <0.0001) for those aged 0–1 years.

The predominant trend among applicants to child development institutes from 2018 to 2022 was their affiliation with the middle socioeconomic ranking. However, notable shifts were observed in the year 2020 in comparison to 2018–2019. Specifically, the proportion of applicants in the middle socioeconomic ranking increased (44.5% vs. 42.6%), the proportion in the lower socioeconomic ranking decreased (31.2% vs. 32.5%), and those in the higher ranking experienced a slight decline (24.3% vs. 25.9%). These variations were found to be statistically significant, with a *p*-value less than 0.0001.

Further, most applicants to child development institutes between 2018 and 2020 had medium to high levels of insurance coverage. However, during the year of closure, there was a substantial decrease in the proportion of applicants with medium-level insurance, from 41.9 to 48.5% in the pre-pandemic period. Meanwhile, the percentage of insured individuals with high-level coverage increased to 49.1% from 42.4%, and those with low-level coverage experienced a slight reduction to 9% from 9.4%. Notably, these differences were found to be statistically significant, with a *p*-value less than 0.0001.

Table 6 presents the number of children at home, their ages (the siblings of the applicant), and the position of the applicant within the family. The majority of applicants to child development centers come from families with an average range of 2.9–2.7 children (with a standard deviation of 1.8–1.7). Notably, during the closure period, applicants originated from families with a statistically significantly lower average number of children (*p*-value<0.0001) than during the pre-COVID years. Nevertheless, from a clinical standpoint, this difference is marginal, amounting to only 0.2%. Further analysis revealed that the number of children in the families of those applying to child development centers during the closures was significantly lower (*p*-value<0.0001). Table 6 illustrates that the predominant position of applicants within the family structure from 2018 to 2022 was as the second child (averaging 2.3–2.2 with a standard deviation of 1.4). However, during the closure period, the average position of the child in the family decreased to 2.2, compared to 2.3 in the years preceding closure. Although this difference was statistically significant (*p*-value<0.0001), its clinical relevance is limited.

**Table 6:** Number of children in the family and position of the applicant child within the family: 2018 to 2022—an analysis using the Mann–Whitney test.

Variable	Year	N	Mean	Std	Median	Minimum	Maximum	*p*-value
Number of kids in family	2018–2019 *N* = 27,036	27,034	2.9	1.8	2.0	1.0	22.0	<0.0001
	2020 *N* = 13,143	13,140	2.7	1.7	2.0	1.0	33.0	
Child order in family	2018–2019 *N* = 27,036	27,035	2.3	1.4	2.0	1.0	15.0	<0.0001
	2020 *N* = 13,143	13,141	2.2	1.4	2.0	1.0	12.0	

## 5 Discussion

This study uniquely examined the effects of the COVID-19 pandemic on the utilization of child development centers by analyzing monthly and annual referral rates, the number of treatments, waiting times (days between diagnosis and treatment initiation), and the prevalence of remote treatments. Additionally, this study assessed the demographic and social characteristics of the applicants.

### 5.1 The number of annual and monthly referrals to child development institutes

The findings of this study revealed a notable decline in both annual referrals during the closure year, particularly during the initial closure period from March to May. This decrease is evident when compared to the years preceding the epidemic (2018–2019), which aligns with the research hypothesis. No prior studies have been published on the rate of referrals to child development centers during the COVID-19 period, either internationally or in Israel. Nevertheless, a similar pattern of reduced referrals has been observed across various urgent and non-urgent medical services for both adults and children in diverse populations and countries. Examples include decreases in neurological procedures, internal consultations, medical center visits, emergency room referrals, and primary medical tests (such as MRI, children’s vaccination, and mammography) during the pandemic compared to corresponding periods (4, 5, 7, 37, 38). A plausible explanation for this decline could be the shift in demographic and social characteristics among applicants between the COVID-19 and pre-COVID period, as supported by previous studies (6, 7, 31) and corroborated by the current study. Notably, during the pandemic, there was a decrease in referrals from individuals with medium- and low-level insurance, coupled with an increase in those with high-level insurance. Additionally, there was an increase in applicants from medium socioeconomic levels, while the rates of applicants from high and low socioeconomic levels decreased.

### 5.2 Diagnosis rate during the review period (2018–2020)

The findings revealed a 10% decrease in the diagnosis rate among all referrals in February 2020, followed by a 30% increase in April 2020. The initial decline in diagnoses in February is likely attributable to the Ministry of Health guidelines that mandated the cancelation of face-to-face treatments and diagnoses, coinciding with a broader shift toward remote treatment methods. Diagnoses, inherently requiring in-person assessment, faced challenges in remote execution, leading to a reduction in February, which was reversed in March–April as the systems adapted. Some treatments swiftly transitioned to remote methods, while others encountered delays due to the technical challenges faced by both parents and treatment staff in adapting to the new systems. By April, technical difficulties eased, resulting in a notable surge in diagnoses as the system stabilized and users became more adept. However, with the systems fully operational in May, there was a return to a normalized situation, and the rate of diagnoses reverted to pre-lockdown levels. Follow-up studies are needed to compare telemedicine diagnosis with treatment during childhood development.

### 5.3 Referral to diagnosis time interval at the institute

The “referral-to-diagnosis” interval calculated as the number of days between the referral date and the diagnosis date (when a qualified specialist provided a confirmed evaluation) has increased across different professions during the COVID-closure year, despite an overall reduction in the number of referrals compared with the pre-COVID period. Due to the lack of prior studies explicitly examining this interval, direct comparison with earlier data is not possible. A plausible explanation for this prolongation is the constrained accessibility of diagnostic services at the Institute for Frontal Diagnostics, influenced by both governmental lockdown directives and internal organizational guidelines. While telemedicine has mitigated delays for ongoing treatments, the diagnostic process itself still necessitates in-person visits, contributing to an extended interval.

### 5.4 Treatment in child development institutes across the years 2018–2020

The overall number of treatments increased notably during the study period. In the years 2018–2019, there were an average of 56,033 treatments annually, whereas in 2020, the figure rose to 89,648 treatments. A direct comparison with previous studies is challenging because no prior research has been published on the number of treatments in child development institutes during the COVID-19 period, both internationally and in Israel. One potential explanation for this increase is the enhanced accessibility facilitated by the organization. The transition to telemedicine and the gradual relaxation of government guidelines likely played crucial roles in providing children or their parents with continued access to health services and the ability to sustain ongoing treatment. This explanation is substantiated by the current findings, indicating a notable 6.9% increase in telemedicine adoption during the closure year of 2020. This trend is consistent with previous research conducted on children (39–42). The use of telemedicine presents numerous advantages not only in times of emergency but also during routine periods, enhancing the accessibility and quality of medical services for infants and children. This is particularly beneficial for geographically and socially marginalized populations (36, 41). However, barriers exist to the widespread adoption of remote treatment methods, including technological challenges, primarily internet accessibility and infrastructure quality. Additional obstacles are linked to patient attitudes and perceptions, including a preference for in-person meetings with therapists and concerns that telemedicine may offer less effective services. Moreover, children face specific challenges such as difficulties in sustaining attention during remote treatment. It is plausible that remote therapy may be unsuitable for children with specific pathologies, such as communication issues, behavioral challenges, attention and concentration difficulties, and delays. Additionally, logistical limitations may pose further constraints, such as cost, preparation time, and organizational approval.

This study presents the findings on the frequency of treatment across various age groups. It is important to note that a direct comparison with previous data is not feasible, given the scarcity of studies that specifically investigated treatments within the age brackets considered in this study. The results indicated a decline in treatment rates for the 0–1 age group during the periods of closure, alongside a marginal increase in the 2–3 and 4–6 age groups compared to the pre-pandemic years. This trend may be attributed to parental concerns about exposing infants to potential infection risks, leading to limited outcomes.

### 5.5 Demographic and social characteristics of applicants to child development institutes during the COVID-19 pandemic

No significant differences in the sex distribution of applicants were observed across the study period. The percentage of male applicants consistently exceeded that of female applicants throughout the study, regardless of the COVID-19 pandemic. However, during the initial quarantine period (March to May 2020), there was a non-significant decrease of 5–12% in the proportion of male applicants compared to females. Unfortunately, no existing studies have conducted a more nuanced comparison of these findings.

The predominant age group for applicants to child development centers was 4–6 years. In the lockdown year, there was a notable decline in applications within this age range, juxtaposed with an upswing in applications from the younger age group of 0–3 years. The literature does not offer conclusive support for the trends in application variations across specific age groups. One plausible explanation for the increased application in the 2–6 age range could be related to potential disruptions in motor development caused by insufficient activity during the closing year. Further research is warranted to delve into the intricacies of the observed shifts in application patterns.

The results of this study revealed a statistically significant (*p* value <0.0001) decline in applicants from both low and high socioeconomic strata during the year of closure (2020), as determined by the locality ranking of their residence. Conversely, there was a statistically significant (*p* value <0.0001) increase in the number of applicants in the middle socioeconomic bracket. The absence of previous comparable findings necessitates a cautious interpretation of these results. The decrease in referrals from low-rated areas during this period may be attributed to logistical challenges, including reduced public transportation availability, difficulties in securing childcare due to school closures, and the reluctance to leave work amid the fear of layoffs. Additionally, the decrease might be linked to a lack of awareness regarding the possibility of accessing services remotely or challenges in using technological means to communicate with institutes. Conversely, individuals in improved socioeconomic situations may have opted for the private sector over the public sector. This choice could be influenced by the flexibility of private services, which may offer treatments either in the patient’s home or in a clinical setting that is deemed quieter and more secure against COVID-19. Support for this hypothesis can be drawn from studies in the United States (6) that highlighted challenges in accessing medical services for individuals with low socioeconomic status across various factors such as race, language, ethnic groups, income levels, and health insurance status in 2020 compared to the pre-pandemic years. Further qualitative follow-up studies are imperative to elucidate the factors influencing the choice of child development centers based on the socioeconomic status of the child’s parents and their residential location.

Most Maccabi Health Fund-insured individuals possess medium-to-high-level insurance coverage. During the year of closure, there was a 6.6% decline in applicants with medium insurance, a 0.4% reduction in applicants with low insurance, and a 6.7% increase in applicants with high insurance. These findings align with those of a prior study from the United States, which reported a decrease in the proportion of applicants with public health insurance during the 2020 Corona period compared with those with private insurance, indicative of a more advanced insurance status, as observed in 2019.

## 6 Limitations

The present study is subject to several limitations. It relied on institute application records, which may register a single child multiple times under different concerns; however, repeat inquiries are rare, occurring perhaps once every few months according to Maccabi Health Services systems. Diagnostic data were incomplete, since not every child underwent evaluation by a developmental pediatrician or received a documented diagnosis. Consequently, only those formally diagnosed by a physician at our center are included in the calculated diagnosis rate. Due to the unavailability of such data, this study does not include information on staffing levels across different professions in child development institutions over time. It is therefore plausible that therapist availability influenced the number of diagnoses and treatments. Note, however, that these data include only applicants to Maccabi Health Services’ child development centers. Although Maccabi is Israel’s second-largest health fund, this does not fully represent all health funds, hospitals, or other centers.

Additionally, an interrupted time series (ITS) analysis could not be implemented because COVID-19–related restrictions and service adaptations unfolded in multiple phases rather than at a single, clearly defined time point, and concurrent modifications to referral pathways and record-keeping practices introduced structural breaks. Moreover, no valid control series existed, since all child development centers were subject to the same restrictions simultaneously. Consequently, we employed Mann–Whitney U tests and chi-square tests for inter-annual and month-to-month comparisons. Future studies with a precisely defined intervention onset and longitudinal data spanning several years before and after the COVID-19 period could use ITS to more accurately isolate temporal effects.

## 7 Conclusion

The 2020 COVID-19 closures led to notable shifts in service utilization patterns and applicant demographics: April referrals spiked, predominantly via remote consultations, while applicants skewed younger, included more middle-SES families, fewer low- and high-SES applicants, and showed increased high-tier insurance coverage. Interpreted through Andersen’s behaviorist and McLeroy’s socioecological frameworks, these trends highlight how familial, community, policy, and organizational factors shaped health-seeking behaviors. Rapid telemedicine adoption bolstered referral continuity and workforce capacity; government outreach and crisis databases improved engagement; and tailored prevention programs enhanced community resilience. To ensure equitable and uninterrupted access during future disruptions, efforts should focus on strengthening telehealth infrastructure, optimizing referral pathways to prevent diagnostic delays, and extending research across diverse child development centers and health funds.

## Data Availability

The data are not publicly available, but can be obtained upon request from the corresponding author.
